# Supplementary material for: Synergy mediates long-range correlations in the visual cortex near criticality
Source: Front Comput Neurosci. 2026 Feb 6;20:1741793. doi: 10.3389/fncom.2026.1741793 (PMC12920449; doi:10.3389/fncom.2026.1741793)
Supplement: Supplementary file 1 [file Data_Sheet_1.pdf]

Supplementary Material

1 DATASET

The dataset involved in the study included 5 spontaneous and 5 stimulated calcium imaging recordings from mouse visual cortex. The details related to number of neurons, field of view, and other parameters of the recordings are shown in the table below (see Table S1).

Subject ID	Context(s)	Duration (min)	Number of Neurons	Frame Rate (Hz)	Field of View (mm)
mouse1	Stimulated	47.91	8592	7.65	3.0 x 3.0
mouse2	Stimulated	39.69	7831	8.98	2.5 x 3.0
mouse3	Stimulated	12.23	4985	7.65	3.0 x 3.0
tigre806b	Spontaneous	40.89	7345	6.95	3.5 x 3.0
tigre812b	Spontaneous	40.80	5885	6.65	3.5 x 3.0
tigre840	Stimulated	46.73	7269	7.65	3.0 x 3.0
tigre840	Spontaneous	41.49	7534	7.65	3.0 x 3.0
tigre847	Spontaneous	39.90	1064	8.49	3.0 x 3.0
tigre869	Stimulated	47.73	5108	7.65	3.0 x 3.0
tigre869	Spontaneous	40.31	4832	7.65	3.0 x 3.0

Table S1. Details of calcium imaging recordings used in the study.

2 CORRELATION DISTRIBUTIONS OVER DISTANCE

In addition to the mean correlation values over distance shown in Figure 4 of the main manuscript, we also analyzed the full distributions of correlation values at different distance ranges. It can be observed from Supplementary Figure S1 that the distributions of correlation values shows a greater spread for the stimulated condition compared to the spontaneous condition, across all the three ranges. This increased variability in correlation values is higher during stimulation, suggests more diverse interactions among neurons when they are actively processing stimuli.

In addition to the distributions, we also plot the average standard deviation of correlation values over distance in Supplementary Figure S2. The standard deviation is consistently higher for the stimulated condition across all distance ranges, further supporting the observation of increased variability in neuronal correlations during stimulation.

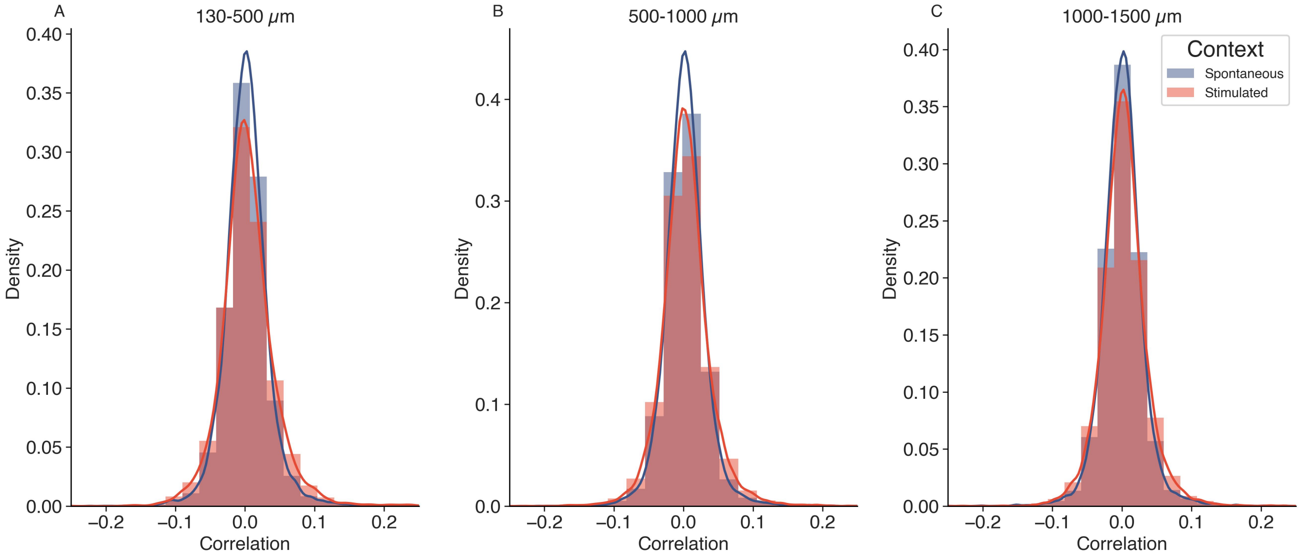

Figure S1. Distributions of correlation values for spontaneous (blue) and stimulated (orange) conditions across three distance ranges: (A) 130-500 µm, (B) 500-1000 µm, and (C) 1000-1500 µm. The histograms illustrate the spread of correlation values, with stimulated conditions showing a wider distribution in all distance ranges.

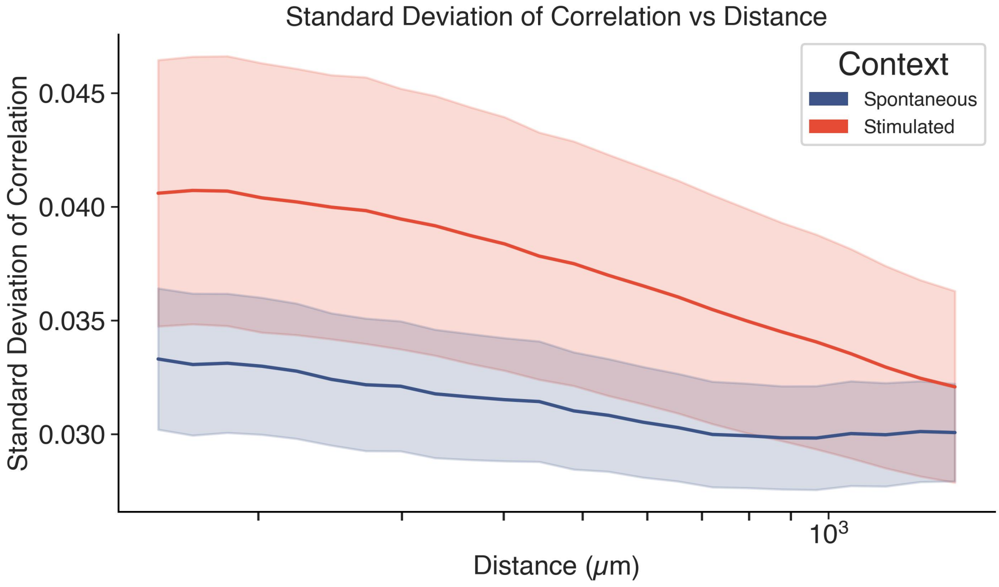

Figure S2. Average standard deviation of correlation values over distance for spontaneous (blue) and stimulated (orange) conditions. The standard deviation is higher for the stimulated condition across all distance ranges, indicating greater variability in neuronal correlations during stimulation.
